# Primary prevention for risk factors of ischemic stroke with *Baduanjin* exercise intervention in the community elder population: study protocol for a randomized controlled trial

**DOI:** 10.1186/1745-6215-15-113

**Published:** 2014-04-09

**Authors:** Guohua Zheng, Bai Chen, Qianying Fang, Hongmei Yi, Qiu Lin, Lidian Chen, Jing Tao, Junzhe Li, Xin Zheng, Moyi Li, Xiulu Lan

**Affiliations:** 1 Academy of Integrative Medicine, Fujian University of Traditional Chinese Medicine, Fuzhou 350122, China; 2 Rehabilitation Medicine College, Fujian University of Traditional Chinese Medicine, Fuzhou 350122, China; 3 Department of Physical Education, Fujian University of Traditional Chinese Medicine, Fuzhou 350122, China; 4 Fujian University of Traditional Chinese Medicine, Fuzhou 350122, China

**Keywords:** *Baduanjin* exercise, Ischemic stroke, Community elder population, Randomized controlled trial

## Abstract

**Background:**

Stroke is a major cause of death and disability in the world, and the prevalence of stroke tends to increase with age. Despite advances in acute care and secondary preventive strategies, primary prevention should play the most significant role in the reduction of the burden of stroke. As an important component of traditional Chinese *Qigong*, *Baduanjin* exercise is a simple, safe exercise, especially suitable for older adults. However, current evidence is insufficient to inform the use of *Baduanjin* exercise in the prevention of stroke.

The aim of this trail is to systematically evaluate the prevention effect of *Baduanjin* exercise on ischemic stroke in the community elder population with high risk factors.

**Methods:**

A total of 170 eligible participants from the community elder population will be randomly allocated into the *Baduanjin* exercise group and usual physical activity control group in a 1:1 ratio. Besides usual physical activity, participants in the *Baduanjin* exercise group will accept a 12-week *Baduanjin* exercise training with a frequency of five days a week and 40 minutes a day. Primary and secondary outcomes will be measured at baseline, 13 weeks (at end of intervention) and 25 weeks (after additional 12-week follow-up period).

**Discussion:**

This study will be the randomized trial to evaluate the effectiveness of Baduanjin exercise for primary prevention of stroke in community elder population with high risk factors of stroke. The results of this trial will help to establish the optimal approach for primary prevention of stroke.

**Trial registration:**

Chinese Clinical Trial Registry: ChiCTR-TRC-13003588.

Registration date: 24 July, 2013.

## Background

Stroke, about 87% being ischemic [1], causes 9% of all deaths around the world and is the second most common single cause of death after ischemic heart disease, with over five million deaths per year globally [2]. Stroke is also the most common cause of disability, with 20% of survivors requiring institutional care after three months and 15 to 30% being permanently disabled [3]. In Europe, it is estimated about that 250,000 people become disabled after their first stroke each year [4]. Stroke consumes about 2 to 4% of total healthcare costs in the world, and accounts for more than 4% of direct healthcare costs in industrialized countries [5]. In China, approximately 1.5 to 2 million new strokes occur each year, and 15 to 30% of them are permanently disabling [6]. The economic burden is estimated at a total annual cost of about ten billion yuan [7]. Although acute treatment is essential in reducing recurrences and disability, primary prevention plays the most significant role in the reduction of the burden of stroke [8]. Therefore, it is important to make efforts to prevent stroke rather than just treat stroke.

Stroke is multi-factorial in causation. Over 100 risk factors, which can be classified as non-modifiable or modifiable, have been identified as likely contributors to its pathogenic progression [9]. The most effective means available for stroke prevention involve modification and treatment of risk factors which mainly include high blood pressure, high cholesterol, high blood glucose, diabetes, certain other cardiac conditions, dyslipidemia, physical inactivity, obesity and so on [10-12]. It has been estimated that over 50% of stroke are preventable through control of modifiable risk factors [13]. For example, lowering blood pressure can reduce an approximate 30 to 40% of the risk of stroke [14,15]; furthermore, 17% further risk reduction may be obtained by reducing the serum concentration of LDL-cholesterol [16]. Nevertheless, despite drug therapies for many of these risk factors being available [17], they remain a limitation because of their high costs, unpredictable side-effects and most subjects having multiple risk factors. Therefore, this task remains a challenge for societies, healthcare and financial systems.

The increasing evidence indicates that exercise and regular physical activity are associated with a reduced risk of stroke through controlling weight and blood pressure, reducing glucose, and modifying lifestyle [18,19], but the precise amounts and type of exercise required to prevent stroke still are unclear [20]. Although intensive forms of physical activity can provide additional protection benefits for stroke, the prevalence of such activities in older people is quite low [21]. As an important component of traditional Chinese *Qigong* exercises, *Baduanjin* exercise is an ancient art and science of healthcare that has been practiced in China for thousands of years [22]. It is a combination of postures, meditation and movements designed to improve holistic health and to achieve the integration of mind and body [23-26]. It can exercise the movable joints and muscle in the whole body, enhancing respiratory function and at the same time modulating mind and spirit [27].

Many studies have demonstrated the beneficial effects of *Baduanjin* exercise on reducing blood lipids [28,29], lowering blood pressure [30], reducing blood sugar and glycosylated hemoglobin [31,32]. Recent review has proven the benefits of *Baduanjin* exercise on cardiopulmonary function and body morphology [33]. Additionally, significant improvement has been reported in balance, strength and flexibility, as well as pain reduction and improvement in sleep quality, psychological well-being and immune function [34-36]. However, few studies are randomized trials, most have significant methodological limitations, and we are not aware of any trials that have evaluated the impact of *Baduanjin* exercise on primary prevention of stroke in the community elder population with high risk factors for stroke.

This study will adopt the strict randomized controlled design. The aim is to systematically evaluate the preventive effect of *Baduanjin* exercise on ischemic stroke in the community elder population with high stroke risk factors by observing the difference between a *Baduanjin* exercise group and a general physical activity group.

## Methods/Design

This is a two-arm, randomized, assessor blinded, parallel controlled trial. The primary aim is to assess the effect of *Baduanjin* exercise on risk factors for stroke in the community elder population.

**Figure 1:**
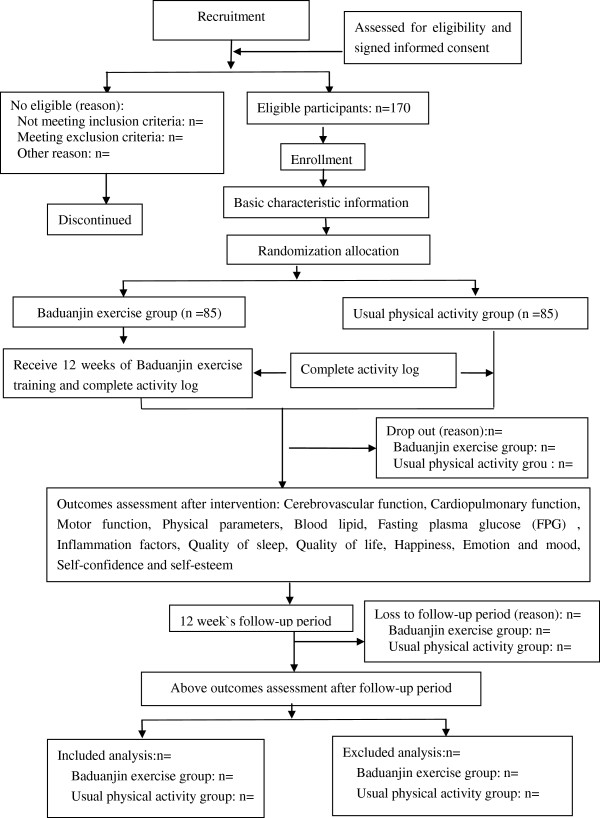
Flow diagram of study design.

This trial will be performed at Wufeng Community Center, in Gulou District, Fuzhou City, China. A total of 170 eligible participants will be randomly allocated into intervention group (the *Baduanjin* exercise group) and control group (general physical activity group) in a 1:1 ratio. The participants in the intervention group will accept a 12-week *Baduanjin* exercise training, at the same time the others in the control group will maintain their original physical activity. Primary and secondary outcomes will be measured at baseline, end of 12-week intervention and after an additional 12-week follow-up period by the assessors who are not involved in this trial at the Affiliated Rehabilitation Hospital, Fujian University of Traditional Chinese Medicine. The statistic analysis will be performed by a statistician at the Center of Evidence Based Medicine, Fujian University of Traditional Chinese Medicine. A flow diagram of this trial is shown in Figure 1.

### Sample size

Sample size computation is based on the changes in cerebral vascular hemodynamic parameter between comparison groups with a 5% per comparison significance level and a two-tailed critical region. The mean and SD of cerebral vascular hemodynamic parameter in the control group as obtained from relevant literature are 32.0 and 6.0 respectively [37]. We expect a 10% mean improvement of this outcome after 12-week *Baduanjin* exercise intervention. A total of 170 participants is calculated as needed to detect the target effect size with 90% power. In order to avoid excessive loss to follow-up, we will illustrate in detail to the participants the potential health benefits of *Baduanjin* exercise. For completeness of whole trial progress we will also provide confidence incentives to participants. Furthermore, we will collect information from participants in both the *Baduanjin* exercise and the usual physical activity control group regarding the likelihood that they will drop out. This information will be used to attempt to reduce the incidence of drop-out.

### Participants and recruitment

#### Eligibility criteria

##### Definition of people with high risk of ischemic stroke

According to ‘The 2012 annual screening and intervention project “Workbook of Stroke High Risk Population”’, the participants with high risk of ischemic stroke should meet two of following items (1) to (7) or item (8): (1) have a history of high blood pressure (systolic/diastolic pressure ≥ 140/90 mmHg), or be taking antihypertensive drugs; (2) have atrial fibrillation; (3) smoke (at least one cigarette each day for one year); (4) have dyslipidemia; (5) have diabetes mellitus; (6) be obviously overweight or obese (BMI ≥ 24 kg/m^2^); (7) have a family history of stroke (a stroke history in three generations); (8) have a history of transient ischemic attack (TIA).

### Inclusion criteria

The eligible participants must meet all the following criteria: (1) be confirmed as people with high risk of ischemic stroke; (2) have not conducted regular physical exercise for at least one year (regular exercise means lasting more than three months with a frequency of three to four times a week and at least 30 minutes per session); (3) be male or female aged from 50 to 70 years old; (4) have signed the informed consent document.

### Exclusion criteria

The eligible participants should not meet any of the following criteria: (1) have a history of stroke; (2) have been suffering from severe cerebrovascular disease, musculoskeletal system diseases, or other sports injury related contraindications; (3) have a communication disorder, such as dementia or mental disorder.

### Recruitment

The participants’ recruitment will be performed at Wufeng Community Center in Gulou district, Fuzhou city. We will recruit the eligible participants through posting-up posters, sending leaflets, and setting a recruiting station in the community center. The potentially eligible subjects who are interested in this study can then contact the recruiters. The eligible subjects will be included if they are in accordance with the inclusion criteria and not meeting any of the exclusion criteria.

### Randomization and allocation concealment

The eligible participants will be randomly allocated in a 1:1 ratio to either *Baduanjin* exercise group or the usual physical activity group. The random allocation sequence will be generated using the statistical software SAS 9.1 by a statistician who will be not involve in this trial at the Center of Evidence Based Medicine, Fujian University of Traditional Chinese Medicine. The random allocation sequence will be managed by the project manager who is not involved in the recruitment program. The eligible participants will be informed of their allocation result by the project manager via telephone after their baseline information has been assessed.

### Blinding

Although it is impossible to blind the participants and exercise coaches due to this being a non-pharmacological intervention trial, two kinds of blind code will be used to blind the outcome assessors and statistician, and will be kept by a project manager who will be involved in the recruitment, intervention, assessment, and statistical analysis procedure of this trial. The participants’ allocation result (the *Baduanjin* exercise group or usual physical activity group) will be replaced by using alphabet ‘A’ or ‘B’ in the first blind code, and the real meaning of ‘A’ or ‘B’ will be marked in the second blind code.

A twice unclosed blind code will be performed in this trial. Firstly, after close of data-base, the project manager will deliver the group code ‘A’ or ‘B’ of participants to the statistician. Secondly, the project manager will declare the real meaning of group ‘A’ or ‘B’ after analysis of all data is completed.

### Intervention

#### Baduanjin exercise group

**Figure 2:**
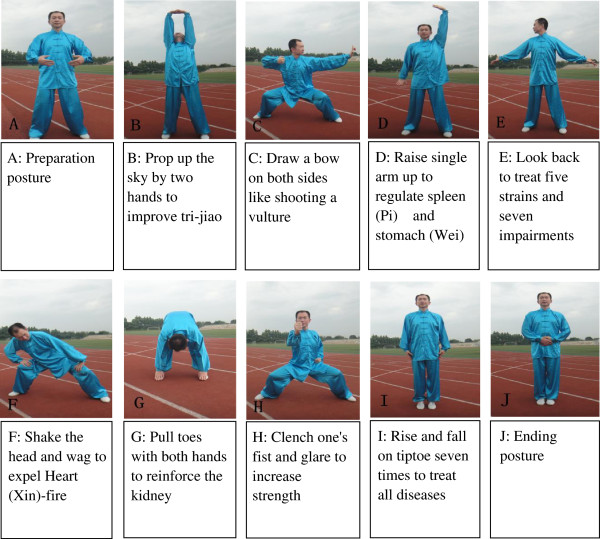
Ten postures of Baduanjin exercise.

The participants in the *Baduanjin* exercise group will receive a 12-week *Baduanjin* exercise training with a frequency of five days per week and 40 minutes per day. Eighty-five participants will be freely gathered into three training units with 25 to 30 people per training unit, and the *Baduanjin* exercise training will be performed at Wufeng Community Center. We will employ three professional coaches, who have engaged in teaching the college students physical education at the Fujian University of Traditional Chinese Medicine for at least five years, to guide participants’ training. The training scheme of *Baduanjin* exercise originated from the ‘*Health Qigong Baduanjin Standard’* enacted by the State Sports General Administration in 2003 [38]. The whole set of Baduanjin exercise consists of ten postures (including the preparation and ending posture) (Figure 2) [39]. Two supervisors will be in charge of the management of each training site to guarantee the quality of Baduanjin exercise training.

### Usual physical activity group

Participants allocated to the control group will not receive any specific exercise training from the study scheme. They will be requested to maintain their original habit of physical activity.

In order to exclude bias from the exceed activity of participants, all participants in both groups will be required to record an activity log in the intervention period, in which the duration and intensity of their activity or exercise in a whole day will be classified into three sections including the duration of low-intensity activity, the duration of moderate-intensity activity, and duration of high-intensity activities.

### Follow-up

After the 12-week intervention period, all participants will enter an additional 12-week unsupervised follow-up period. They will resume their original lifestyle in the follow-up period, but they will be required to record their activity log as in the intervention period. Two supervisors will investigate the quality of each activity log by telephone once every two weeks.

### Outcome assessment

The outcome assessment will be conducted at baseline (-2 to -1 weeks), end of intervention (13 weeks), and end of follow-up period (25 weeks).

The variables in this trial consist of basic characteristic variables, primary outcomes, and secondary outcomes. The basic characteristic variables will be measured at baseline (before randomized allocation); the primary or second outcomes will be measured at baseline, end of intervention period, and end of follow-up period, respectively. All outcome assessment will be independently performed by the assessors or operators who are blinded the allocation results of participants. A summary of all measurements in this trial is shown in Table 1.

**Table 1:** Study flow chart

**Items**		**Before enrollment (weeks)**	**Intervention period (weeks)**	**End of the exercise (weeks)**	**Follow-up (weeks)**	**End of the follow-up (weeks)**
		**−2 to (-1)**	**1 to 12**	**13**	**13 to 24**	**25**
Recruitment		×				
Enrollment		×				
Inclusion criteria		×				
Exclusion criteria		×				
Informed consent		×				
Basic characteristic variables		×				
Randomization and allocation concealment		×				
Primary outcomes	Cerebrovascular function	×		×		×
	Cardiopulmonary function	**×**		**×**		**×**
Secondary outcomes	Motor function	**×**		**×**		**×**
	Physical parameters	**×**		**×**		**×**
	Blood lipids	**×**		**×**		**×**
	Fasting plasma glucose(FPG)	**×**		**×**		**×**
	Inflammation factors	**×**		**×**		**×**
	Quality of sleep	**×**		**×**		**×**
	Quality of life	**×**		**×**		**×**
	Happiness	**×**		**×**		**×**
	Emotion and mood	**×**		**×**		**×**
	Self-confidence and self-esteem	**×**		**×**		**×**
Adverse events recorded			×		×	
Activity log			×		×	

### Basic characteristic variables

Demographic characteristics will be collected by the recruiters through using a standardized questionnaire (gender, age, nationality, education level, occupation, marital status).

Information on stroke risk factors regarding smoking, drinking, height (cm), weight (kg), blood pressure, family history of stroke, blood lipids, and blood sugar will be gathered through medical records.

Other information including history of diseases, physical activity or exercise habits, and drug administration information will be collected by the recruiters through using a standardized questionnaire.

### Primary outcomes

Cerebrovascular function consists of cerebral vascular hemodynamic and cerebrovascular elasticity. Cerebral vascular hemodynamic parameters consist of the maximum blood flow velocity (Vmax), minimum blood flow velocity (Vmin) and mean blood flow velocity (Vmean). Those parameters from the vertebral, basilar, middle cerebral, anterior cerebral and posterior cerebral arterial territories of the brain will be measured using Color Doppler ultrasound imaging device (PHILIPS, product type: IU22) by the professional operators at the Affiliated Rehabilitation Hospital of Fujian University of Traditional Chinese Medicine. Cerebrovascular elasticity consists of the elasticity index (PI) and vascular resistance index (RI) of vertebral, basilar, middle cerebral, anterior cerebral and posterior cerebral arteries. It will be measured using Color Doppler ultrasound imaging device (PHILIPS, product type: IU22) by the professional operators at the Affiliated Rehabilitation Hospital of Fujian University of Traditional Chinese Medicine.

### Secondary outcomes

Cardiopulmonary function consists of static lung function and cardiac function. Static lung function will be measured using the cardiopulmonary function instruments (JAEGER, Germany, product type: OXYCON PRO PC) by the professional operators at the Affiliated Rehabilitation Hospital of Fujian University of Traditional Chinese Medicine. Cardiac function will be measured using the Color Doppler ultrasound imaging device (product type: SIEMENS Acuson X300) by the professional operators at the medical examination center of Second People’s Hospital of Fujian Province.

Motor function includes lumbar and lower limb proprioception function and balance function. Lumbar and lower limb proprioception function will be measured using the Pro*-K*in proprioception evaluation and training system (produced by Tecnobody S.r.l, Dalmine, Italy, product type: PK254P) by the professional operators at the Affiliated Rehabilitation Hospital, Fujian University of Traditional Chinese Medicine. Balance control will be measured using the Pro*-K*in proprioception evaluation and training system (produced by Tecnobody S.r.l, Dalmine, Italy, product type: PK254P) by the professional operators at the Affiliated Rehabilitation Hospital, Fujian University of Traditional Chinese Medicine.

Physical parameters include body mass index (BMI, weight(kg)/height(m)^2^), waist-to-hip ratio (waist circumference (cm)/hip circumference (cm)), and flexibility. BMI will be measured using body scale by the assessors in this trial. Waist-to-hip ratio will be measured using a metric rule by the assessors in this trial. Flexibility will be measured through ‘sit and reach’ test using a ‘sit and reach’ tester produced by Zhongtitongfang Co., Ltd., Beijing*,* China (product type: CSTF-TQ-5000) by the assessors in this trial.

Blood lipid index consists of serum total cholesterol (TC), serum triglyceride (TG), low- density lipoprotein (LDL) and high-density lipoprotein (HDL). They will be measured by the operators at the Affiliated Rehabilitation Hospital, Fujian University of Traditional Chinese Medicine.

Fasting plasma glucose (FPG) will be measured by the operators at The Affiliated Rehabilitation Hospital, Fujian University of Traditional Chinese Medicine.

Inflammation factors including serum homocysteine (Hcy), serum c-reactive protein, IL-6, and TNF-α will be measured using the ELISA method by the assessors at Center Medical Laboratory, Fujian University of Traditional Chinese Medicine.

Quality of sleep will be measured through use of the Pittsburgh Sleep Quality Index (PSQI) [40]. The PSQI is a self-rated questionnaire which assesses sleep quality within a one month timeframe. Nineteen individual items generate seven dimensions: subjective sleep quality, sleep latency, sleep duration, habitual sleep efficiency, sleep disturbances, use of sleeping medication, and daytime dysfunction [41]. The Chinese version of PSQI has been reported by Liu Xianchen and his colleagues [42] to have acceptable internal consistency, test-retest reliability, construct validity, and criterion-related validity.

Quality of life will be measured through use of the Medical Outcomes Study (MOS) item short form health survey, SF-36 [43]. The SF-36 scale was constructed to survey health status in the MOS. The SF-36 includes one multi-item scale that assesses eight health concepts: limitation in physical activities because of health problems; limitations in social activities because of physical or emotional problems; limitations in usual role activities because of physical health problems; bodily pain; general mental health; limitation in usual role activities because of emotional problems; vitality; and general health perceptions [44]. The Chinese version of SF-36 has been reported by Jin Wen-zheng and his colleagues [45] to have reliability including split-half reliability, internal consistency, criterion validity and structure validity.

Happiness will be measured by use of the Memorial University of Newfoundland Scale of Happiness (MUNSH) [46]. The Chinese version of MUNSH has been reported by Wang Wen-Xin and his colleagues [47] to have better effect of re-measure of measuring table, intrinsic correlation by Hotelling’s *t*-test, better reliability in homogeneity and better reliability.

Emotion and mood will be measured through use of the Brief Profile of Mood State (BPOMS) [48]. The BPOMS is proven to be an excellent measure of affective mood state fluctuation in a wide variety of populations with a stronger reliability and validity. Six factors assessed identifiable mood or affective states include tension-anxiety, vigor-activity, depression-dejection, fatigue-inertia, anger-hostility, and confusion-bewilderment [49]. The Chinese version of BPOMS has been reported by Song Chi and his colleagues [50] to have acceptable internal consistency, construct validity, and criterion-related validity.

Self-confidence and self-esteem will be measured by use of the Rosenberg Self-Esteem Scale (RSES) [51]. The RSES is a self-esteem measure widely used in social-science research. The ten items, five of the items having positively worded statements and five having negatively worded ones, assess the subject’s level of self-esteem by asking the respondents to reflect on their current feelings [52]. The Chinese version of RSES has been reported by Xiangdong Wang and his colleagues [53] to have acceptable internal consistency, construct validity, and criterion-related validity.

### Safety

Although no adverse event from *Baduanjin* exercise is reported to this day, all unexpected adverse events during the intervention period will be reported to the project manager, and the causality with *Baduanjin* exercise intervention will be analyzed. If serious adverse events do occur, they will be reported to the primary researchers and ethics committee immediately and they will decide whether the participant needs to withdraw from this trial.

### Statistical analysis

Statistical analyses will be performed using SPSS 21.0 (IBM, Chicago, IL, USA) software packages by a statistician, who is not involved in the outcome measurements, at Center of Evidence Based medicine, Fujian University of Traditional Chinese Medicine. The statistical significance is defined as two-sided *P* value of < 0.05. In descriptive analysis of the sample, the central tendency for the continuous variables will be expressed using mean (standard deviation) or median (interquartile range) for symmetrical distributions; normality will be tested using the Kolmogorov-Smirnov test. Appropriate transformations will be applied in cases of a non-normal distribution. The categorical variables will be expressed as proportions with their standard error. Statistical comparisons for the baseline characteristics, primary and secondary outcomes between groups will be compared using the Student’s *t-*test or Mann-Whitney *U*-test for continuous variables and the Pearson Chi-squared or Fisher exact test for categorical variables. Analysis of the primary and secondary outcomes will be on the basis of the intention-to-treat (ITT) population and per-protocol (PP) population. The result of the ITT analysis will be compared with that of the PP analysis to determine whether the results are consistent. Every effort will be made to minimize missing data and drop-out or loss to follow-up during the study period. Missing data will be filled by Bayesian methods. If the amount of missing data for primary outcome or potential confounders is substantial (that is > 10%), multiple imputation methods will be considered to perform the statistic analysis. Linear models or linear regression will be applied for dependent continuous variables and logistic regression models for dependent categorical variables if incomparability of baseline characteristics between groups appears. Subgroup analysis stratified by participants’ sex will be used for the primary outcomes. Analysis of variance (ANOVA) will be used for the repeated measurement data, and the *post hoc* comparison will be applied if the difference is found to be significant. Adverse events will be listed and analyzed using a Chi-squared or Fisher’s exact test. Severe adverse events will be listed in detail.

### Ethics

This protocol is in accordance with the Declaration of Helsinki. The study protocol and consent forms were approval by Medical Ethics Committee of the Affiliated People's Hospital, Fujian University of Traditional Chinese Medicine (approval number: 2013-021-02). Participants will sign the informed consent document prior to participation.

### Dissemination policy

Study protocol has been registered, and is available on the Chinese Trial Registry website (Registered in ChiCTR.org with the identifier ChiCTR-TRC-13003588). All participants will be the first to be informed of trail results. They will then be published in scientific journals if possible regardless of the magnitude or direction of effect. No professional writers will be employed.

## Discussion

*Baduanjin* exercise is a traditional Chinese *Qigong* which is characterized by simple, slow, relaxing movements. It has been practiced as a popular and safe community exercise to promote health for hundreds years in China [54]. *Baduanjin* exercise involves eight sections of movement, each of which benefits different parts of the body [38]. Previous studies have indicated that *Baduanjin* exercise can improve blood lipid metabolism, insulin sensitivity, and blood pressure for community older adults [29-31]. However, few studies are randomized trials and most have significant methodological limitations [32,36]. Current evidence therefore, is insufficient to inform the use of *Baduanjin* exercise in the prevention of stroke. The primary purpose of this trial was to systematically assess the effectiveness of regular *Baduanjin* exercise on primary prevention of stroke in community elder population through detection of improvement in cerebrovascular function, cardiopulmonary function, and exposure of risk factors for stroke compared with people with usual physical activities. This trial will employ adequate methods to reduce bias, such as randomization, blinding to the outcome assessors and statistical analyzers, a large subject population and analysis according to the intent-to-treat (ITT) principle. It is expected that *Baduanjin* exercise will have an obvious positive effect on primary prevention of ischemic stroke in the community elder population with high risk factors.

This trial has potential limitations. In the ideal situation, everyone involved in a randomized controlled trial should be blinded but this is not always feasible in non-pharmacological trials [55]. Although the participants and exercise coaches will be not blinded in this trial, the outcome assessors and statistical analyzers will be blinded concerning information about treatment allocation. In the intervention period, all participants in both groups will be required to record their activity log, which includes the duration and intensity of their activity or exercise in a whole day. In the process of outcomes assessment, the participants will be asked not to reveal their treatment group to assessors. The whole intervention of *Baduanjin* exercise in the treatment group will be performed in an outdoor setting, so occasional intervention interruption may therefore be unavoidable due to adverse weather. In this does occur, we will ask participants in the treatment group to perform self-practice at home.

In summary, this study will be the first randomized trial to evaluate the impact of *Baduanjin* exercise on primary prevention of stroke in a community elder population with high risk factor of stroke. The results of this trial will help to establish the optimal approach for preventing stroke in high risk groups and provide reliable evidence for its application in the rehabilitation of Traditional Chinese Medicine.

## Trial status

Recruitment started while the manuscript was being finished.

## Abbreviations

SD: Standard deviation; BMI: Body mass index; TIA: Transient ischemic attack; SAS 9.1: Statistical analysis system 9.1; SPSS 21.0: Statistic package for social science 21.0; TC: Total cholesterol; TG: Serum triglyceride; LDL: Low- density lipoprotein; HDL: High-density lipoprotein; FPG: Fasting plasma glucose; Hcy: Serum homocysteine; IL-6: Interleukin 6; TNF-α: Tumor necrosis factor α; ELISA: Enzyme-linked immuno sorbent assay; PSQI: Pittsburgh sleep quality index; MOS: Medical outcomes study; SF-36: Item short form health survey; MUNSH: Memorial University of Newfoundland Scale of Happiness; BPOMS: Brief Profile of Mood State; RSES: Rosenberg self-esteem scale; ITT: Intention-to-treat; PP: Per-protocol; ANOVA: Analysis of variance.

## Competing interests

The authors declare that they have no competing interests.

## Authors’ contributions

CLD, TJ, and ZGH conceived of the study, designed the study protocol, and drafted the manuscript. CB wrote the manuscript and participated in the coordination and implementation of the study. ZGH revised study protocols and wrote several sections of the manuscript. TJ is in charge of coordination and direct implementation. YHM, LQ, FQY, LJZ, ZX, LMY and LXL helped to develop the study measures and analyses. All authors contributed to drafting the manuscript and have read and approved the final manuscript.
